# The Effect of Bariatric Surgery on the Relation Between Retinol-Binding Protein 4 (RBP4) and Vitamin D Plasma Levels in Male Obese Population

**DOI:** 10.7759/cureus.32733

**Published:** 2022-12-20

**Authors:** Essa J Faqihi, Khalid Alregaiey, Mohammed A Altuwayjiri, Mohammad N Alamri, Bader A Alshehri, Muhammad Iqbal

**Affiliations:** 1 Physiology, King Saud University, Riyadh, SAU

**Keywords:** retinol-binding protein 4 (rbp4), saudi arabia, obesity, bariatric surgery, vitamin d

## Abstract

Objectives

Obesity is viewed as a risk factor for several life-threatening diseases. Bariatric surgeries are regarded as a safe and effective way of treating morbid obesity and are associated with alterations in molecules such as Vitamin D and retinol-binding protein 4 (RBP4). The aim of this study was to examine an association between Vitamin D and RBP4 in healthy obese individuals undergoing gastric sleeve surgery.

Methods

In this observational retrospective cohort study, we used clinical and biochemical profiles of morbidly obese healthy male subjects (BMI > 38.3) who underwent laparoscopic sleeve gastrectomy. RBP4 levels were evaluated by ELISA before and 6-12 months after surgery. Data were analysed using SPSS 23 statistical software.

Results

RBP4/Vitamin D ratio was reduced significantly after surgery (p < 0.001), however, there was no correlation between Vitamin D and RBP4 (p = 0.353). BMI was reduced significantly after surgery (p < 0.001) but was not correlated with RBP4 or Vitamin D levels before and after surgery (p > 0.05). A significant increase in the levels of calcium post-surgery was observed (p< 0.001).

Conclusions

In conclusion, plasma levels of Vitamin D were not correlated with RBP4 before or after laparoscopic gastric sleeve surgery.

## Introduction

Over the last three decades, obesity’s prevalence has increased in a lot of countries all over the world. Obesity is defined by a BMI of 30 or higher. Solid evidence regarding the increase in heart failure and mortality incidence in relation to obesity and adiposity has been reported [1]. Lifestyle modifications including physical activity and dietary intervention are considered first-line therapy. Nevertheless, they have limited efficacy in morbid or severe obesity. Therefore, for morbidly obese individuals or obesity with comorbidity, bariatric surgeries are a better treatment option. Bariatric surgery has increasing popularity for being the most effective treatment for morbidly obese individuals. This is credited to the considerable weight loss experienced and total improvement in comorbidities related to obesity. Still, due to the complications that might arise postoperatively, it is typically deemed as the last option for treatment [2]. It is estimated that 15,000 bariatric surgeries are done yearly in Saudi Arabia [3]. Patients must go through an appropriate evaluation of nutrients, including micronutrient measurement before any bariatric surgical procedures according to the American Society for Metabolic & Bariatric Surgery guidelines. The risk of developing postoperative complications has been linked to the non-compliance of bariatric patients to dietary recommendations [4].

Retinol-binding protein 4 (RBP4) is an adipokine and is produced by adipocytes and hepatocytes, and it is a specific transporter protein for retinol serum. Several studies document that the RBP4 level was elevated in type 2 diabetes mellitus, obesity, and other diseases that are resistant to insulin [5]. RBP4 concentrations are markedly increased in morbidly obese patients, this elevation is believed to be caused by the large mass of adipocytes that secrete that protein [6]. Vitamin D deficiency is a common complication of bariatric surgeries, especially malabsorptive ones [1]. Because of nutritional deficiencies, obese people might have low serum levels of Vitamin D even before the surgery given that Vitamin D levels and BMI have been shown to have an inverse relationship, which may be due to altered dietary habits, avoidance of sun exposure, reduced bioavailability of Vitamin D due to its sequestration on adipose tissue, and decreased hepatic 25-hydroxylase in obese patients with non-alcoholic fatty liver disease [7-9].

This is aggravated by the decrease in Vitamin D’s bioavailability due to its sequestration in the additional adipose tissue [10]. Vitamin D function on adipokines is a current research hotspot. Evidence has reported that Vitamin D is correlated with leptin, adiponectin, and other adipokines, but not many studies have concentrated on analysing the relationship between RBP4 and Vitamin D. Metheniti et al. had shown that the level of Vitamin D was low in ultra-obese young females and was associated largely with RBP4 and neutrophil gelatinase-associated lipocalin (NGAL) [11]. In another study, the results showed that Vitamin D was associated negatively with RBP4 and the concentration of RBP4 decreased drastically after intake of Vitamin D supplements [5].

To the extent of our knowledge, data are scarce reporting alterations in plasma levels of RBP4 and Vitamin D before and after bariatric surgery [5]. Therefore, the present study aimed to assess the correlation between plasma levels of RBP4 and Vitamin D in male obese patients following gastric sleeve surgery.

## Materials and methods

This observational retrospective cohort study was conducted by using data from a previous study [12]. In the present study, we analysed the association between RBP4 and Vitamin D plasma levels in 33 obese patients before and after gastric sleeve surgery. All male patients, 25-50 years of age who had a BMI >35 kg/m^2^, attended the surgery clinic for bariatric surgery (gastric band or sleeve gastrectomy), were healthy (were not taking any medication) and provided consent to participate were included in the study. Female patients, those aged below 25 or above 50 years, having BMI <35 kg/m^2^, didn’t undergo bariatric surgery (gastric band or sleeve gastrectomy), and did not consent to participate were excluded. Moreover, patients taking any medication or having any of the following conditions such as liver dysfunction, renal dysfunction, uncontrolled diabetes, cardiovascular disease (CVD), known growth hormone disorders such as dwarfism or acromegaly, and known genetic disorders such as Down syndrome were also excluded.

Briefly, approximately 5 ml blood was taken from obese patients before and 6 months after gastric sleeve surgery following overnight fasting, in tubes with and without anticoagulant (sodium citrate) to process complete blood count (CBC), lipid profile, and molecular profile. RBP4 was analysed by using a simple step human ELISA kit (AB 108897) following the manufacturer’s instructions (Abcam, Cambridge, UK). The study was approved by Institutional Review Board, College of Medicine, King Saud University (Ref. No. 20/0825/IRB). Data were analysed using SPSS 23 statistical software. Descriptive statistics (mean, standard deviation) were used to describe the demographic and baseline data of the study group. Student’s t-test for paired sample was used to compare the mean values of pre and post-surgery. The Pearson correlation coefficient was used to quantify the relationships between quantitative variables. A p-value of ≤ 0.05 and 95% confidence intervals were taken as statistically significant.

## Results

The mean age of the study sample was 35 years, and the preoperative weight was 150 kg (±25). Vitamin D levels were significantly increased after bariatric surgery (from 8.5±4.8 to 20.0±9.7 (nmol/L), (mean±SD), p < 0.001, 95% CI (7.8, 15.3), Table 1.

**Table 1:** Difference in plasma levels of RBP4, Vitamin D, RBP4/Vitamin D ratio and BMI before and after surgery (n = 33, df = 32). RBP4: retinol-binding protein 4

	Pre-surgery Mean(±SD)	Post-surgery Mean(±SD)	Mean difference (±SD)	95% CI	t value	p-value
RBP4 (ng/ml)	4382.9 (±231.8)	4393.3 (±190.4)	10.4 (±264.3)	(-83.3, 104.1)	0.227	0.82
Vitamin D (nmol/L)	8.5 (±4.8)	20.0 (±9.7)	11.5 (±10.5)	(7.8, 15.3)	6.342	< 0.001
RBP4/Vitamin D ratio	666.9 (±328.5)	283.8 (±170.2)	-383.1 (±329.5)	(-499.9, -266.3)	-6.679	< 0.001
BMI*	52.2 (±9.7)	40.1 (±8.9)	-12.1 (±6.8)	(-14.6, -9.6)	-9.885	< 0.001

There were no differences in levels of RBP4 before and after surgery (from 4382.9 ±231.8 to 4393.3 ±190.4 ng/mL, (mean±SD), p = 0.82, 95% CI (-83.3, 104.1), Table 1. BMI was decreased significantly after surgery (from 52.2±9.7 to 40.1±8.9 kg/m^2^ (mean±SD), p < 0.001, 95% CI (-14.6, -9.6), Table 1.

**Figure 1:**
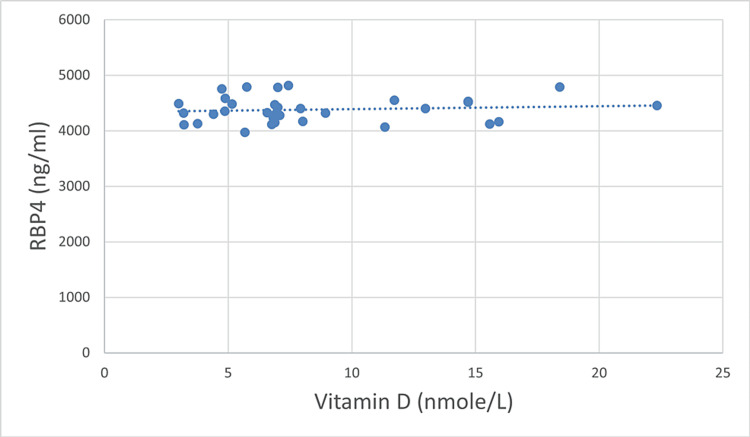
Correlation between plasma levels of RBP4 and Vitamin D before surgery (n = 33). RBP4: retinol-binding protein 4

**Figure 2:**
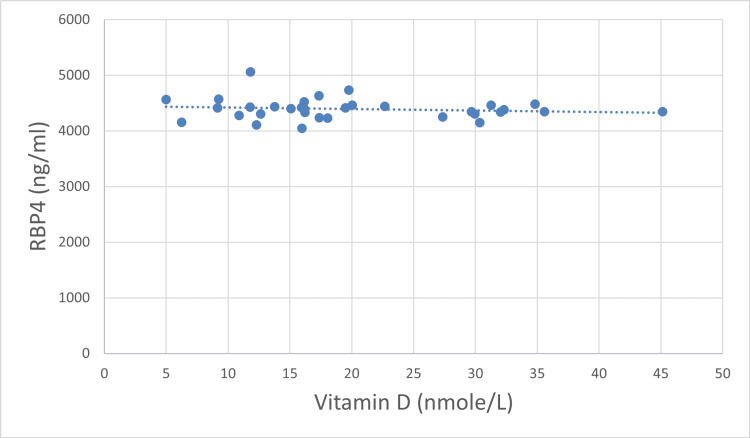
Correlation between plasma levels of RBP4 and Vitamin D after surgery (n = 33). RBP4: retinol-binding protein 4

We did not find any statistically significant correlation between RBP4 and Vitamin D plasma levels either pre- (p = 0.56) or post-bariatric surgery (p = 0.448), Table 2. However, the direction of these minor relations was different before and after surgery. Before surgery, RBP4 and Vitamin D were in direct relation, while after bariatric surgery they were inversely related (Table 2, Figures 1-2), although not significantly.

**Table 2:** Correlation between plasma levels of RBP4 and Vitamin D pre- and post- surgery (n = 33). RBP4: retinol-binding protein 4

Bariatric Surgery	Person Correlation Coefficient	Direction	p-value
Pre-	0.105	+	0.56
Post-	0.137	-	0.448

There was no significant correlation between changes in RBP4 and Vitamin D levels after surgery (p = 0.353, Table 3).

**Table 3:** Correlation between change in plasma levels of RBP4 and Vitamin D after bariatric surgery. RBP4: retinol-binding protein 4

	Person Correlation Coefficient	Direction	p-value
Changes in RBP4 and Vitamin D	0.167	+	0.353

We calculated the ratio between RBP4 and Vitamin D levels to get the cumulative effect of surgery on the relation between these two markers. There was a reduction in the RBP4/Vitamin D ratio (from 666.9 ±328.5 to 283.8±170.2, (mean±SD), p = <0.001, 95% CI (-499.9, -266.3), Table 4. However, there was no correlation of BMI with RBP4 and Vitamin D levels before and after surgery (p > 0.05, Table 4).

**Table 4:** Correlation between patients' BMI with plasma levels of RBP4, Vitamin D, and RBP4 to Vitamin D ratio (n = 33). RBP4: retinol-binding protein 4

BMI			r	Direction	p
Preoperative	RBP4	Preoperative	0.082	+	0.651
		Postoperative	0.042	-	0.814
		Changes	0.102	-	0.571
	Vitamin D	Preoperative	0.022	-	0.905
		Postoperative	0.199	+	0.266
		Changes	0.195	+	0.277
	preoperative RBP4/Vitamin D ratio		0.21	+	0.24
Postoperative	RBP4	Postoperative	0.012	+	0.950
		Changes	0.114	+	0.541
	Vitamin D	Postoperative	0.094	+	0.613
		Changes	0.151	+	0.419
	postoperative RBP4/Vitamin D ratio		0.028	-	0.883
Changes	Changes in RBP4 levels		0.140	+	0.452
	Changes in Vitamin D levels		0.325	-	0.074
	Changes in RBP4/Vitamin D ratio		0.003	+	0.987

There was a significant increase in the levels of calcium post-surgery (from 2.16 ±0.1 to 2.51±0.13, (mean±SD), p = <0.001), Table 5.

**Table 5:** Difference between pre-surgery and post-surgery calcium levels of male obese patients (n = 33, df = 32).

Item	Pre-surgery		Post-surgery		P-value
	Mean	SD	Mean	SD	
Calcium	2.16	0.1	2.51	0.13	< 0.001

## Discussion

The coexistence of obesity with abnormalities in carbohydrate and lipid metabolism has been well documented. However, the exact mechanisms linking obesity with metabolic disorders still remain to be elucidated in detail [13]. The objective of our study was to investigate a correlation between RBP4 and Vitamin D plasma levels in male obese patients before and after bariatric surgery. The main findings of our study were a statistically significant increase in Vitamin D levels and a reduction in the RBP4/Vitamin D ratio after surgery. But there was no statistically significant correlation between plasma levels of RBP4 and Vitamin D either before or after bariatric surgery.

RBP4 is a globular protein, mainly produced by the liver and adipose tissue. The primary role of this molecule is to transport vitamin A (retinol) in circulation. RBP4 has been proposed to be implicated in the pathophysiology of the metabolic consequences of obesity [14]. There is a decrease in RBP4 levels with a hypocaloric diet and weight loss achieved by lifestyle or weight loss achieved by sleeve gastrectomy (SG) bariatric surgery [15]. A study by Wang et al. reported a significant decrease in RBP4 levels after laparoscopic sleeve gastrectomy in patients of Chinese ethnicity [16]. We have previously shown that RBP4 levels were not changed after bariatric surgery in the adult Saudi male population. Age-wise distribution of study participants showed decreased RBP levels in older individuals, but not to a significant level [12].

As a metabolic risk factor in obesity, RBP4 has been associated with insulin resistance and adipose accumulation. RBP4 levels are elevated in obesity and are positively associated with BMI. Our study showed no statistically significant relationship between BMI, RBP4, and Vitamin D before and after surgery and the changes in their levels. There was no relationship between BMI and the RBP4/Vitamin D ratio. However, a study by Wang et al. reported an association between higher RBP4 levels and BMI in obese patients [16].

Vitamin D in obese individuals is thought to be stored in body fat compartments, resulting in decreased serum bioavailability and circulatory deficiency. Vitamin D stimulates the metabolism of fatty acids and suppresses lipogenesis [11]. Recent literature has suggested that the pro-inflammatory state linked with obesity may lead to alterations in Vitamin D metabolism and lowered circulating levels [17]. Furthermore, chronic kidney disease, which is more prevalent in obese individuals, is associated with Vitamin D deficiency. Adiposity and BMI have been shown to have a negative correlation with serum Vitamin D levels. Thus, bariatric surgical patients tend to be Vitamin D deficient [18]. There is evidence that typically over 50% of bariatric surgical patients have low Vitamin D levels, and in some cases, over 90% [19].

There have been apprehensions that bariatric surgery might cause or worsen Vitamin D deficiency in obese patients, possibly due to proposed reasons that include malabsorptive effects of the procedures and decreased oral intake. Vitamin D is crucial for the homeostasis of calcium and bone metabolism. In our study, a statistically significant increase in Vitamin D levels occurred after surgery. Our findings are consistent with another study which reported an increase in Vitamin D levels 12 months after surgery and had a significant inverse association between BMI and Vitamin D levels, our findings may be influenced by the Vitamin D supplementation that was prescribed to all participants during the study period [20]. This was corroborated by a significant positive correlation between % total weight loss (TWL) and Vitamin D levels. Our study showed a weak negative association between changes in BMI and the changes that occurred in the levels of Vitamin D, but the association was not statistically significant (p = 0.074).

We did not find any statistically significant correlation between levels of RBP4 and Vitamin D either before (p = 0.56) or after surgery (p = 0.448) and changes in the plasma levels of RBP4 and Vitamin D (p = 0.353). When the study participants were divided into two age groups (25-35 and 35-50 years of age), the results were non-significant. Metheniti et al. had shown a positive correlation between RBP4, Vitamin D, and lipocalin-2 in obese female children and adolescents [11]. In another study, Vitamin D was associated negatively with RBP4, and the concentration of RBP4 decreased drastically after the intake of Vitamin D supplements [5]. However, there are no studies showing a correlation between Vitamin D and RBP4 after gastric sleeve surgery and need further investigations.

The present study showed a significant increase in the levels of calcium post-surgery (p< 0.001). Fox et al. revealed that 8.5% of all patients had low adjusted calcium levels at baseline [20]. The hypocalcaemia rates increased over time, with no significant differences between procedures or sexes. Patients with hyperparathyroidism or Vitamin D insufficiency/deficiency had higher rates of hypocalcaemia.

## Conclusions

In summary, there was no significant association between RBP4 with Vitamin D plasma levels in healthy obese male patients after gastric sleeve surgery. Future studies should be conducted with a larger sample size on a broader scale of patients’ age and weight, including women, with various follow-up time intervals following gastric sleeve surgery. Moreover, since our study indicated no significant correlation between Vitamin D and RBP4 (p = 0.353), further studies are needed to confirm this and ascertain its potential impact in routine clinical practice.
